# Characteristics of traditional Chinese medicine users and prescription analysis for pediatric atopic dermatitis: a population-based study

**DOI:** 10.1186/s12906-016-1158-1

**Published:** 2016-06-08

**Authors:** Yu-Chun Chen, Yi-Hsuan Lin, Sindy Hu, Hsing-Yu Chen

**Affiliations:** Department of Medical Research and Education, National Yang-Ming University Hospital, I-Lan, Taiwan; Institute of Hospital and Health Care Administration, School of Medicine, National Yang-Ming University, Taipei, Taiwan; Division of Chinese Internal Medicine, Center for Traditional Chinese Medicine, Chang Gung Memorial Hospital, No. 123, Dinghu Rd., Gueishan Dist., Taoyuan City, 33378 Taiwan; Graduate Institute of Clinical Medical Sciences, College of Medicine, Chang Gung University, Taoyuan, Taiwan; School of Traditional Chinese Medicine, College of Medicine, Chang Gung University, Taoyuan, Taiwan; Department of Dermatology, Chang Gung Memorial Hospital, Taoyuan, Taiwan; Department of Cosmetic Science, Chang Gung University of Science and Technology, Taoyuan, Taiwan

**Keywords:** Traditional Chinese medicine, Atopic dermatitis, Pediatrics, Chinese herbal medicine

## Abstract

**Background:**

Atopic dermatitis among children is an important issue due to relapses and skin manifestations. Traditional Chinese medicine (TCM) is commonly used to treat children with atopic dermatitis. The aim of this study was to investigate the characteristics and TCM prescriptions of patients with atopic dermatitis using a nationwide database.

**Methods:**

Children younger than 12 years of age diagnosed with atopic dermatitis, defined as ICD-9-CM codes 691.8 and 692.x, were identified from the database. Data on age, diagnosis codes, area of residence and use of corticosteroids of the TCM users were recorded. Association rule mining was used to analyze the prescriptions used for atopic dermatitis.

**Results:**

We identified 13,646 children with atopic dermatitis using TCM in 2007. Female gender (OR: 0.83 for male gender), adolescence (OR: 10.0, 95 % CI: 8.88–11.15) and allergic rhinitis (OR: 2.44, 95 % CI: 2.10–2.85) were associated with the use of TCM. Fewer of the TCM users were prescribed with corticosteroids (35.8 % of all TCM users), but the TCM users had a higher rate of long-term topical corticosteroid therapy (10.6 % for TCM users versus 2.0 % for those who did not use TCM). Chinese herbal medicine (CHM) was used by 93.7 % of all TCM users in 36,398 prescriptions. On average, 5.6 kinds of CHM were used in combination. The relationship between the CHMs constituted a network, in which Xiao-Feng-San was the core treatment for atopic dermatitis.

**Conclusions:**

In this study, we described the characteristics of children with atopic dermatitis who use TCM in Taiwan. and identified the core CHM treatment. Further research on the safety and efficacy of this treatment are still needed.

## Background

Atopic dermatitis is a chronic inflammatory skin disease which is highly prevalent among children [1, 2]. The clinical features of atopic dermatitis vary with age and the stage of disease, such as oozing, erosions and eroded vesicles in the acute stage, and hyper-pigmented lichenification of the skin in the chronic stage. Severe skin itching is a major complaint, especially at night, during the entire course of atopic dermatitis, as well as insomnia and chronic fatigue, all of which substantially affect the children’s quality of life [1, 3]. The pathogenesis of atopic dermatitis is complicated and not fully understood. Genetic factors, skin barrier disorders, immune dysregulation, mast cell activation and skin infections have all been reported to be highly associated with atopic dermatitis [4–7]. Multiple management strategies have been proposed to control symptoms, such as emollients containing urea, anti-histamines, corticosteroids, anti-microbial agents and even immunotherapy [1, 8]. Of these strategies, corticosteroids remain the most important, and are used in both topical and systemic form, however the use of corticosteroids is often limited due to concerns over adverse effects [9–11].

Traditional Chinese medicine (TCM) is commonly used to control the symptoms of atopic dermatitis among children in Taiwan, and Chinese herbal medicine (CHM) is one of the most commonly used modalities of TCM treatment [12–14]. Although many studies have reported on the use and effectiveness of CHM, a detailed analysis of the prescription patterns of CHM for the treatment of atopic dermatitis is lacking. Therefore, the characteristics of the children who use TCM and which CHMs are given to these children in daily clinical practice are unknown.

This study aimed to identify the use pattern of TCM for pediatric atopic dermatitis and the most commonly used CHMs. In addition to the characteristics of the TCM user, we aimed to identify the most important CHMs and CHM combinations, and thereby a network of these CHMs. Such information would be a good reference for clinicians and also provide good candidates for further studies, since TCM doctors usually prescribe several CHMs in combination to treat a disease.

## Methods

### Data source

The data used in this study were obtained from the National Health Insurance Research Database (NHIRD) in Taiwan. This database is particularly useful for epidemiological studies due to the high coverage rate of the National Health Insurance (NHI) program in Taiwan (>98.3 % of the total population) and full reimbursement for TCM including CHM, acupuncture and manipulation therapy [14]. The NHIRD contains detailed information on the patients’ demographic features, diagnosis and treatment. International Classification of Diseases, ninth edition, Clinical Modification (ICD-9-CM) codes are used to verify the diagnosis of each visit, and up to three ICD-9-CM codes can be used for each visit. The first ICD-9-CM code is the primary reason for the visit, and the reliability of ICD-9-CM coding has been well validated [15, 16]. The NHIRD has been used as the data source for many studies on TCM epidemiology and prescription analysis [14, 17].

### TCM prescriptions

The details of TCM prescriptions are recorded in the NHIRD, which is made available for research purposes. In addition to acupuncture and manipulation therapy, every component of CHM is recorded in this database. In Taiwan, two kinds of CHM are reimbursed, herbal formulas (HFs) and single herbs (SHs). Each HF contains SHs with fixed proportions strictly based on the TCM classics, and each HF can be combined with other HF or SH. All CHMs in Taiwan are manufactured at pharmaceutical factories governed by the Good Manufacturing Practice guidelines, and all pesticides and heavy metals are strictly prohibited.

### Study design and subjects

**Fig. 1:**
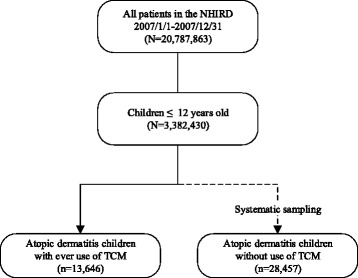
Flow diagram of this study

The flow diagram of study enrollment is demonstrated in Fig. 1. Patients with a diagnosis of atopic dermatitis (ICD-9-CM codes: 691.8 and 692.x) who were aged 0–12 years were enrolled in this study. TCM users were defined as those who used at least one form of TCM from 2007/1/1 to 2007/12/31, and TCM nonusers were defined as those who never used TCM. A TCM users are retrieved from the entire database, and TCM nonusers are collected by systemically sampling from the entire database, which represents the general population since there was no difference in gender or age between sampled and whole database [18]. Diagnosis of atopic dermatitis was mainly based on clinical manifestations such as itching, scratching, oozing, erythema and lichenification over skin lesions. The age of the enrolled subjects was limited to 12 years in this study since atopic dermatitis is highly prevalent before adolescence [19]. The subjects with missing data were excluded from the final analysis.

### Study variables

To investigate differences between those who did and did not use TCM, we used gender, age, type and number of combined allergic diseases, area of residence, level of urbanization and use of corticosteroids as covariates with odds ratios. Co-morbidities were identified on the basis of ICD-9-CM codes: 477.0, 477.1, 477.2, 477.8 and 477.9 for allergic rhinitis, 493.x for asthma and 491.x for chronic bronchitis. Because the features of atopic dermatitis are closely related to where the patients live and the urbanization level, both geographical location and urbanization level were used as covariates [20]. In Taiwan, urbanization level can be divided into seven levels, in which level 1 is the most urbanized and level 7 is the least. In this study, level 4 to level 7 were combined as level 4, as the number of children living in these areas was relatively small [14]. We also assessed differences in the use of corticosteroids between those who did and did not use TCM, since corticosteroids are the mainstay of therapy for atopic dermatitis, and the use of corticosteroids has been reported to be an influential factor for the use of alternative therapy [21]. To identify the utilization pattern of corticosteroids for each patient, the use rate, duration and frequency of prescriptions of corticosteroids in the previous 1 year were analyzed. The average daily dose and prevalence of CHM are used to describe the use of commonly used CHMs.

### Bias assessment

As we used a nationwide design in this study, the selection and referral bias were largely minimized, since the NHI covers almost all everyone in Taiwan. In addition, these data were more complete than hospital-based data. Furthermore, registration bias was largely eliminated by using ICD-9-CM codes to identify diseases, of which the reliability has been proven.

### Statistical analysis

We used descriptive statistics to describe the features of the TCM users. Independent t-tests and chi-square tests were used to examine differences in continuous and categorical variables between those who did and did not use TCM, respectively. We used logistic regression analysis to differentiate the characteristics between those who did and did not use TCM, and adjusted odds ratios (aORs) to adjust covariates. In addition, to analyze the TCM prescriptions, we used association rule mining (ARM). ARM is a well-developed data mining technique to explore the relationships between two items in a large dataset, and it has been successfully used for CHM prescription analysis [17]. The ARM algorithm has previously been described in detail [22]. We used the open-source software “R” with the “arule” package to analyze TCM prescriptions, and the commercial SAS software package to analyze the characteristics of TCM users. All results with a two-tailed *p*-value of less than 0.05 were considered to be statistically significant.

## Results

### Characteristics of the TCM users

Between 2007/1/1 and 2007/12/31, 13,646 children attended a TCM clinic for atopic dermatitis. We also enrolled 28,457 patients who did not use TCM from database as the control group. The demographic characteristics of the TCM users are demonstrated in Table 1. There were slightly more female TCM users, and slightly more male TCM nonusers (OR: 0.83 for males, 95 % CI: 0.79–0.86, *p* < 0.001). In addition, the use of TCM was more common in the older children than in the younger children (OR: 10.0 among the school ages children, 95 % CI: 8.88–11.15, *p* < 0.001), and most of the TCM users were school-aged children (69 % of all TCM users). This trend was more prominent in the TCM users than in the nonusers. Furthermore, the children with more co-existing allergic diseases tended to use TCM more (1.34 times more than the TCM nonusers). The use of TCM was 2.44 times more common among the children with atopic dermatitis and allergic rhinitis (95 % CI: 2.10–2.85). In contrast, the children with both atopic dermatitis and respiratory problems were unlikely to use TCM. In addition, the children living in more urbanized areas used TCM more frequently.

**Table 1:** Demographic features of traditional Chinese medicine (TCM) users for pediatric atopic dermatitis in Taiwan in 2007

Demographic features	TCM users *n* = 13,646	TCM nonusers *n* = 28,457	aOR	95 % CI	*p*-value
Sex					
Female (%)	6973 (51.1)	13,292 (46.7)	1		
Male (%)	6673 (48.9)	15,165 (53.3)	0.83	0.79–0.86	<0.001
Age (mean (SD))	7.81 (3.4)	5.00 (3.7)			<0.001
Infants, <1 (%)	348 (2.6)	4986 (17.5)	1		
Toddlers, 1–2 (%)	1230 (9.0)	6396 (22.5)	2.78	2.45–3.16	<0.001
Pre-schools, 3–5 (%)	2657 (19.5)	6036 (21.2)	5.57	4.94–6.29	<0.001
School ages, 6–12 (%)	9411 (69.0)	11,039 (38.8)	10.0	8.88–11.15	<0.001
Combined allergic diseases					
Asthma (%)	1381 (10.1)	2057 (7.2)	0.87	0.77–0.99	0.041
Allergic rhinitis (%)	4565 (33.5)	3877 (13.6)	2.44	2.10–2.85	<0.001
Bronchitis (%)	4604 (33.7)	12,220 (42.9)	0.53	0.45–0.62	<0.001
Number of combined allergic diseases					
0	6293 (46.1)	14,604 (51.3)	1		
1	4774 (35.0)	10,418 (36.6)	1.33	1.14–1.55	<0.001
≥ 2	2579 (18.9)	3435 (12.0)	1.34	1.02–1.90	0.039
Geo-location					
Northern area	6144 (45.0)	14,191 (49.9)	1		
Middle area	3624 (26.6)	5413 (19.0)	1.77	1.67–1.89	<0.001
South area	3648 (26.7)	8281 (29.1)	1.12	1.06–1.19	<0.001
East area	230 (1.7)	572 (2.0)	1.03	0.87–1.22	0.718
Urbanization level					
1 (most urbanization)	4218 (30.9)	8586 (30.2)	1		
2 (more urbanization)	4418 (32.4)	8411 (29.6)	1.05	0.99–1.11	0.109
3 (middle urbanization)	2306 (16.9)	4855 (17.1)	0.90	0.83–0.96	<0.001
4 (least urbanization)	2704 (19.8)	6605 (23.2)	0.75	0.70–0.81	<0.001

*Abbreviations*: *aOR* adjusted odds ratio, *CI* confidence interval

### Use of corticosteroids among the TCM users

Only 35.8 % of the TCM users had used corticosteroids before using TCM, compared to more than two-thirds of the TCM nonusers (Table 2). The use of topical corticosteroids was most common, and most of the children received treatment for less than 14 days. Nevertheless, more TCM users received long-term (more than 14 days) topical corticosteroid therapy. In addition, even though about 10 % of the children received systemic corticosteroids for atopic dermatitis in both groups, long-term systemic corticosteroid use was still higher among the TCM users (Table 2).

**Table 2:** Differences in use of corticosteroid use among TCM users and nonusers of pediatric atopic dermatitis

Utilization patterns of corticosteroid	TCM users *n* = 13,646 (%)	TCM nonusers *n* = 28,457 (%)	*p*-value
Exposure to steroids			
Any steroids	4886 (35.8)	19,334 (67.9)	<0.001
Topical steroids	4763 (34.9)	18,062 (63.5)	<0.001
Systemic steroids	1235 (9.1)	2807 (9.9)	0.004
Prescribed duration of steroids			
Topical steroids			<0.001
Never used	8883 (65.1)	10,397 (36.5)	
Short term (<14 days)	3315 (24.3)	17,492 (61.5)	
Long term (≥14 days)	1448 (10.6)	568 (2.0)	
Systemic steroids			<0.001
Never used	12,411 (90.9)	25,650 (90.1)	
Short term (<14 days)	906 (6.6)	2730 (9.6)	
Long term (≥14 days)	329 (2.4)	77 (.3)	
Frequency of visits for steroids			
Topical steroids			<0.001
0	8883 (65.1)	10,395 (36.5)	
1–3	3807 (27.9)	18,060 (63.5)	
≥ 4	956 (7.0)	2 (.0)	
Systemic steroids			<0.001
0	12,411 (90.9)	25,650 (90.1)	
1–3	1068 (7.8)	2807 (9.9)	
≥ 4	167 (1.2)	- (.0)	

### CHMs commonly used to treat the children with atopic dermatitis

**Fig. 2:**
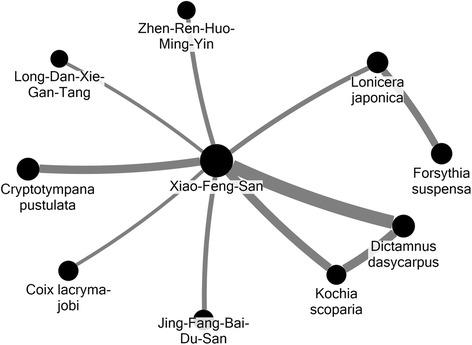
CHM network of top ten CHM combinations for pediatric atopic dermatitis. *Larger circle means higher prevalence (5–32 %) and thicker connection line means higher prevalence of connected CHM

CHM was the most commonly used form of TCM for the children with atopic dermatitis. Overall, 12,790 children (93.7 %) used CHM with a total of 36,398 prescriptions in 2007. More than 600 kinds of CHM were used during this period, and an average of 5.6 kinds of CHM were used in combination in one prescription. Xiao-Feng-San (XFS) was the most commonly used HF (31.6 % of all prescriptions), followed by Jing-Fang-Bai-Du-San (10.6 %) and Xin-Yi-Qing-Fei-Tang (8.9 %) (Table 3). *Glycyrrhiza uralensis* was the most commonly used SH (16.7 %), followed by *Dictamnus dasycarpus* (13.4 %) and *Cryptotympana pustulata* (13.3 %) (Table 4). The most commonly used CHM combination was XFS with *Dictamnus dasycarpus* (6.7 %), and the relationships between the commonly used CHMs constituted a CHM network (Fig. 2). This network clearly demonstrated that XFS was the core CHM for treating atopic dermatitis among children for two reasons: XFS was the most commonly used single CHM, and, more importantly, in order to achieve the efficacy, XFS was needed for other CHMs in combinations.

**Table 3:** The top 5 commonly prescribed herbal formulas (HF) for pediatric atopic dermatitis during 2007. (Total prescriptions = 36,398)

Herbal formulas	Ingredients	Dosage (gm/day)	Number of prescriptions (%)	
Xiao-Feng-San (XFS)	*Saposhnikovia divariata,*	3.4	15,676	(31.6)
	*Atractylodes lancea,*			
	*Schizonepeta tenuifolia,*			
	*Arctium lappa,*			
	*Glycyrrhiza uralensis,*			
	*Rehmannia glutinosa,*			
	*Gypsum Fibrosum,*			
	*Clematis armandii*			
	*Anemarrhena asphodeloides*			
	*Angelica sinensis*			
	*Cryptotympana pustulata*			
	*Sesamum indicum*			
	*Sophora flavescens*			
Jing-Fang-Bai-Du-San	*Notopterygium incisum,*	2.9	3841	(10.6)
	*Angelica biserrata*			
	*Ligusticum chuanxiong*			
	*Bupleurum chinense*			
	*Peucedanum praeruptorum*			
	*Citrus aurantium*			
	*Platycodon grandiflorum*			
	*Panax ginseng*			
	*Poria cocos*			
	*Glycyrrhiza uralensis.*			
Xin-Yi-Qing-Fei-Tang	*Magnolia biondii*	3.0	3254	(8.9)
	*Scutellaria baicalensis*			
	*Gardenia jasminoides*			
	*Ophiopogon japonicus*			
	*Lilium brownii*			
	*Gypsum Fibrosum*			
	*Anemarrhena asphodeloides*			
	*Glycyrrhiza uralensis*			
	*Eriobotrya japonica*			
	*Cimicifuga heracleifolia*			
Zhen-Ren-Huo-Ming-Yin	*Lonicera japonica,*	3.0	2803	(7.7)
	*Saposhnikovia divariata,*			
	*Angelica dahurica,*			
	*Angelica sinensis,*			
	*Paeonia lactiflora,*			
	*Boswellia carterii,*			
	*Commiphora myrrha,*			
	*Fritillaria thunbergii,*			
	*Trichosanthes kirilowii,*			
	*Gleditsia sinensis,*			
	*Citrus reticulata,*			
	*Glycyrrhiza uralensis*			
Long-Dan-Xie-Gan-Tang	*Gentiana scabra*	2.7	2540	(7.0)
	*Scutellaria baicalensis*			
	*Gardenia jasminoides*			
	*Alisma plantago-aquatica*			
	*Caulis Clematidis armandii*			
	*Plantago asiatica*			
	*Angelica sinesis*			
	*Rehmannia glutinosa*			
	*Bupleurum chinense*			
	*Glycyrrhiza uralensis*

**Table 4:** The top ten commonly used single herb (SH) for pediatric atopic dermatitis (Total prescription = 36,398)

Name	Dosage (gm/day)	Number of prescriptions (%)	
*Glycyrrhiza uralensis*	0.9	6076	(16.7)
*Dictamnus dasycarpus*	0.9	4876	(13.4)
*Cryptotympana pustulata*	0.9	4846	(13.3)
*Lonicera japonica*	1.1	4204	(11.6)
*Forsythia suspensa*	1.1	4141	(11.4)
*Coix lacryma-jobi*	1.2	3588	(9.9)
*Paeonia suffruticosa*	1.1	3520	(9.7)
*Kochia scoparia*	0.9	3461	(9.5)
*Angelica dahurica*	1.4	2234	(6.1)
*Schizonepeta tenuifolia*	1.0	2,186	(6.0)

## Discussion

To the best of our knowledge, this is the first large-scale study to report differences in the characteristics of children with atopic dermatitis who did and did not use TCM. TCM was more commonly used for older and female children, especially those with allergic rhinitis and multiple allergic diseases compared to the children who never used TCM. Although the age at onset of atopic dermatitis is usually around 5 years, the higher use of TCM among older children may reflect poor control of atopic dermatitis, in that the parents of the children are more willing to try TCM when they are older. Dissatisfaction with current western medical treatment may be the reason for the use of TCM in the older children, and this trend is similar to that reported in the general population [14, 21].

The children with more allergic diseases tended to use TCM therapy, especially when they had both atopic dermatitis and allergic rhinitis. This may also explain why the children living in more urbanized areas tended to use TCM, as allergic diseases tend to be more prevalent in these area [23]. The use of western medical treatment significantly increased when the children had more co-morbid allergic diseases, such as increasing doses of anti-histamines or the combined use of different kinds of anti-histamines and even corticosteroids. The potentially higher risk of adverse events with higher doses of western medicine may cause parents to consider TCM. In contrast, it has been reported that the use of herbal medicine can improve sneezing and skin itching with just one prescription with minimal side effects [24]. Unlike disease-specific treatments from a western medicine viewpoint, TCM doctors usually take the whole human body into consideration when making prescriptions, and try to control symptoms by using a single prescription [25].

Moreover, the use of corticosteroids was quite different between the TCM users and nonusers. Overall, the use of corticosteroids was lower among the TCM users before they received TCM treatment, however the long-term use was relatively higher than in the TCM nonusers. The adverse effects of corticosteroids often concern parents, such as skin atrophy and telangiectasia with the long-term use of topical corticosteroids, and endocrine disorders with the use of systemic corticosteroids [9–11]. Thus, concerns over the use of corticosteroids is one of the most important factors for choosing TCM to control symptoms [21]. The influence of this factor may be strengthened by dissatisfaction with regards to standard western medical therapy. Children who visit TCM doctors tend to have more severe symptoms, even if they are receiving western medical treatment, and therefore seek add-on TCM therapy to improve symptom control [26].

CHM is the most commonly used TCM to treat atopic dermatitis, and more than 90 % of all TCM users had used CHM in this study. This trend is similar to urticaria, which is also an allergic skin disease with frequent relapses [17]. In contrast to topical agents as the mainstay therapy in western medical treatment, all CHMs are taken orally. From a TCM point of view, the main goals of treatment are to modify the patient’s constitution and correct immunity disorders, not only to control local disease [27]. Therefore, it is believed that the treatment effect can be enhanced by using topical and oral agents concurrently to relieve skin discomfort from inside to outside of the body. Under this treatment model, patients’ symptoms can be well-controlled and the use of corticosteroids can be reduced, as observed in small-scale clinical trials [13, 24, 28].

XFS is the core CHM treatment for pediatric atopic dermatitis due to its high prevalence and multiple relationships with other CHM. XFS is a well-known CHM used for chronic erythematous and itching skin lesions [17]. The effectiveness of XFS was shown in one clinical trial, and several potential pharmacological mechanisms have been reported, including inhibition of IgE secretion, anti-inflammation, anti-oxidation and immuno-modulation for a Th1/Th2 imbalance [28–31]. XFS provides comprehensive coverage of the pathogenesis of atopic dermatitis, and therefore it has become the core treatment for atopic dermatitis. The role of XFS can be clearly seen in the CHM network (Fig. 2). Several other CHMs are commonly combined with XFS to enhance the effectiveness of XFS, including *Dictamnus dasycarpus* and *Kochia scoparia* which may enhance the anti-inflammatory and anti-allergic effects of XFS, and *Cryptotympana pustulata* which may enhance the anti-oxidation and anti-inflammatory effects of XFS [32–35]. The combination of CHMs is not only used to enhance the existing effects of XFS, but also to complement XFS. The use of *Lonicera japonica* and *Forsythia suspense* may add anti-microbial effects to XFS, which are thought to be beneficial to control atopic dermatitis [36–38]. Investigations on CHMs and their combinations provide important information about candidates for further studies, since the CHM prescriptions used to treat atopic dermatitis may be very different between use in daily practice and those detailed in TCM textbooks [39].

There are both strengths and limitations to this study. By using a nationwide database, potential referral bias and patient selection bias can be minimized, since all medical records for children with atopic dermatitis can be obtained. In addition, due to high coverage of this database, the results of this study can be regarded as a kind of consensus of TCM doctors and thus be a references to both TCM doctors and researchers. Nevertheless, the use of folk medicine was not included in this study, and therefore the prevalence of TCM use may be underestimated. Folk medicine in Taiwan is not reimbursed by the NHI and is not strictly regulated. This study focused on the use of CHM only, and thus the results are reliable and repeatable since all information on such CHM is made public in Taiwan. Another limitation of this study is the lack of information on CHM for different subtypes, or “zheng” in Chinese, of atopic dermatitis, since the subtypes of each patient were not available in the NHIRD.

## Conclusion

The children with atopic dermatitis and more co-morbidities (especially allergic rhinitis) and those living in more urbanized areas were associated with a higher use of TCM. CHM was the most common type of TCM used for children with atopic dermatitis, and XFS was the core treatment. Further studies on the efficacy and safety of such treatment are warranted.

## Abbreviations

ARM, association rule mining; CHM, Chinese herbal medicine; HF, herbal formula; ICD-9-CM, International Classification of Diseases, ninth edition, Clinical Modification; NHI, National Health Insurance; NHIRD, National Health Insurance Research Database; OR, odds ratio; SH, single herb; TCM, traditional Chinese medicine; XFS, Xiao-Feng-San
